# Areas with High Hazard Potential for Autochthonous Transmission of *Aedes albopictus*-Associated Arboviruses in Germany

**DOI:** 10.3390/ijerph15061270

**Published:** 2018-06-15

**Authors:** Stephanie Margarete Thomas, Nils Benjamin Tjaden, Christina Frank, Anja Jaeschke, Lukas Zipfel, Christiane Wagner-Wiening, Mirko Faber, Carl Beierkuhnlein, Klaus Stark

**Affiliations:** 1 Department of Biogeography, University of Bayreuth, 95447 Bayreuth, Germany; Nils.Tjaden@uni-bayreuth.de (N.B.T.); Anja.Jaeschke@uni-bayreuth.de (A.J.); Lukas.Zipfel@uni-bayreuth.de (L.Z.); Carl.Beierkuhnlein@uni-bayreuth.de (C.B.); 2 Robert Koch Institute, 13353 Berlin, Germany; FrankC@rki.de (C.F.); FaberM@rki.de (M.F.); StarkK@rki.de (K.S.); 3 Baden-Württemberg Health Authority, 70565 Stuttgart, Germany; christiane.wagner-wiening@rps.bwl.de

**Keywords:** *Aedes albopictus*, Asian tiger mosquito, species distribution model, global change, vector-borne diseases, mosquito-borne diseases, chikungunya, dengue, Europe

## Abstract

The intensity and extent of transmission of arboviruses such as dengue, chikungunya, and Zika virus have increased markedly over the last decades. Autochthonous transmission of dengue and chikungunya by *Aedes albopictus* has been recorded in Southern Europe where the invasive mosquito was already established and viraemic travelers had imported the virus. *Ae. albopictus* populations are spreading northward into Germany. Here, we model the current and future climatically suitable regions for *Ae. albopictus* establishment in Germany, using climate data of spatially high resolution. To highlight areas where vectors and viraemic travellers are most likely to come into contact, reported dengue and chikungunya incidences are integrated at the county level. German cities with the highest likelihood of autochthonous transmission of *Aedes albopictus*-borne arboviruses are currently located in the western parts of the country: Freiburg im Breisgau, Speyer, and Karlsruhe, affecting about 0.5 million people. In addition, 8.8 million people live in regions considered to show elevated hazard potential assuming further spread of the mosquito: Baden-Württemberg (Upper Rhine, Lake Constance regions), southern parts of Hesse, and North Rhine-Westphalia (Lower Rhine). Overall, a more targeted and thus cost-efficient implementation of vector control measures and health surveillance will be supported by the detailed maps provided here.

## 1. Introduction

The dengue, chikungunya, and Zika fevers are emerging viral diseases of significant global public health concern [1,2,3,4,5]. Transmission of these diseases requires the presence of competent vectors and viraemic humans or primates. In non-endemic areas, infected travellers returning from endemic countries can start the chain of infection if vectors are present and environmental conditions are appropriate.

The invasive vector mosquito *Aedes albopictus*, closely associated with human settlements [6,7], is now established in large areas of Southern Europe [8]. The low-temperature-tolerant mosquito [9] is a known or suspected vector for more than 20 arboviruses [10,11] for which often neither vaccinations nor specific antiviral treatments are available [7]. Autochthonous transmissions of dengue (DENV) and chikungunya virus (CHIKV) by *Ae. albopictus* were recently recorded in Southern Europe for the first time (Table 1).

The hazard potential of autochthonous transmission now applies to Germany, with at least four factors coming together. Firstly, *Ae. albopictus* continues to spread further north and into Germany. While the species has been present in the Mediterranean region since 1979 [8], it took years for it to move towards more temperate climates. First found in 2007 in southwestern parts of Germany in the state of Baden-Württemberg [21], larvae were discovered in 2011 at the Czech–Austrian border [22] and in Austria in the Inn valley in 2012 [23]. In 2014, *Ae. albopictus* populations were found in the Upper Rhine Valley in south-western Germany near Freiburg, suggesting locally occurring reproduction of the mosquito [24]. Further single-specimen findings on parking lots near motorways confirm the repeated introduction of this species by the long-distance transport from Southern Europe over the German–Austrian and German–Swiss borders [25,26]. A sharp increase in the number and size of detected *Ae. albopictus* populations along motorways was recorded in the summers of 2015 and 2016 in the German states of Baden-Württemberg, Hesse, and Rhineland Palatinate [27]. With the recent discoveries of overwintering populations in Freiburg, Heidelberg, and Jena [28,29], the species must be considered to be established in Germany.

Secondly, German infrastructure for mosquito surveillance, monitoring, and control is severely under-developed in large portions of the country. While the *Kommunale Aktionsgemeinschaft zur Bekämpfung der Schnakenplage* (KABS, the German Mosquito Control Association) has been performing mosquito control along the Upper Rhine since 1976, similar organizations do not exist in other parts of the country (see Figure S2). Larger-scale surveillance and monitoring projects like the recent CuliMo (a country-wide monitoring project coordinated by the Friedrich-Loeffler-Institute running since 2015) only receive funding for limited amounts of time.

Thirdly, the global intensity and extent of transmission of arboviruses such as DENV, CHIKV, and Zika virus (ZIKV) has increased markedly [2,30,31], and global travel is rapidly expanding [32]. Travel between Germany and tropical areas where these viruses are endemic continues to increase (the number of passengers arriving in Germany from tropical countries increased by 23% from 2011 to 2016 [33]). This trend increases the probability and frequency of the presence of viraemic returnees in Germany [34].

Fourthly, at least in some areas of Germany, summer conditions may already be suitable for vector-borne transmission of DENV and CHIKV. For ZIKV, the local transmission potential of *Ae. albopictus* is less clear under current German conditions, but may increase with rising temperatures [11]. Most outlooks on the effects of climate change in Germany signal increasing temperatures in decades to come.

In order to implement appropriate infection prevention measures and to be able to quickly react to developing situations, areas where such autochthonous transmissions may occur need to be identified. Here, we model the current and future climatically suitable regions for *Ae. albopictus* establishment in Germany on the basis of current data on the European occurrence of *Ae. albopictus*, using climate data of spatially high resolution. Reported DENV and CHIKV incidences at the county level are then combined with climate suitability for vector establishment to highlight areas where vector and viraemic travellers are most likely to come into contact. This identification of current areas showing a hazard potential supports public administrations to effectively plan vector control measures and health surveillance to avoid autochthonous transmission of *Aedes*-associated arboviruses in Germany.

## 2. Materials and Methods

**Figure 1:**
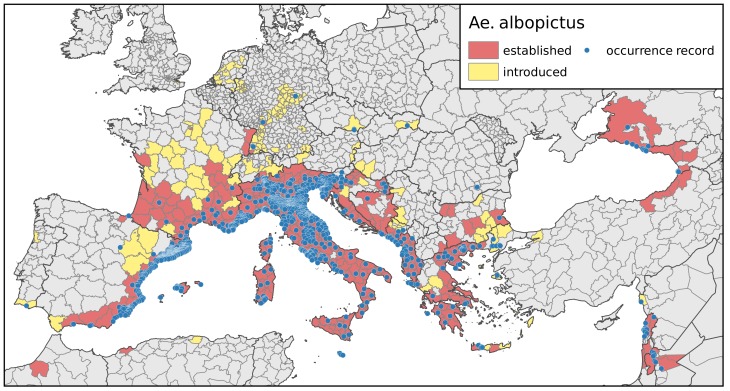
European distribution of *Aedes albopictus* as of January 2018. Blue dots: high-precision occurrence records derived from the literature and used for modelling (*n* = 1336). Red and yellow areas: administrative units with established populations and introduced specimens, respectively, according to the European Centre for Disease Prevention and Control [38]. Areas are level-3 administrational units following the nomenclature des unités territoriales statistiques (nomenclature of territorial units for statistics, NUTS) as used by the European Union. For Germany, this corresponds to the district (Kreis) level.

Estimation of the potential spatial distribution of vector species is an essential step for assessing areas potentially affected by a vector-borne disease [35]. Here, we used European occurrence records of *Ae. albopictus* to calibrate our models in order to project the environmental niche of the invasive species within the European environment as accurately as possible. Records on the presence of *Ae. albopictus* at the European scale were taken from Kraemer et al. [36]. Additionally, scientific articles and reports of mosquito surveillance published between 1979 (the year *Ae. albopictus* was first discovered in Europe [37]) and January 2018 were scanned for additional records of infestations. Records where no long-term establishment of populations (specimens found over at least 2 years or overwintering otherwise suggested) could be inferred were discarded, resulting in a total of 1336 observed records (Figure 1).

Bioclimatic variables with a spatial resolution of 2.5 arcmin (≈5 km) were taken from the global climatic dataset worldclim, comprising 19 variables [39]. Out of these 19 variables a pre-selection of variables based on expert knowledge on the ecology of *Ae. albopictus* was carried out. Here we mainly focused on upper and lower environmental limits (maximum and minimum temperature) as well as temperature and precipitation periods (e.g., mean temperature of warmest quarter, precipitation of driest quarter). With the remaining eleven variables we conducted hierarchical partitioning (R package “hier.part”, version 1.0-4) [40] to assess the influence of the single variables and to further reduce the set of variables to the most important ones. Six variables remained after the hierarchical partitioning and were used for modelling: annual mean temperature, minimum temperature of the coldest month, mean temperature of the warmest quarter, mean temperature of the coldest quarter, precipitation of the driest month, and precipitation of the driest quarter.

The model of the current and future climatic suitability of *Ae. albopictus* was based on four different model algorithms: the generalized boosted model (GBM), the generalized additive model (GAM), maximum entropy (maxent), and random forest (RF). All model runs were performed using the biomod2 package (version 3.3-7) [41] implemented in R (version 3.4.2) [42]. The current European distribution was best depicted by the GBM (area under the curve (AUC): 0.98, total sum of squares (TSS): 0.86). Subsequently, only the GBM was used to project the potential future distribution. We used observed occurrences and randomly generated pseudo-absences within Europe where the number of pseudo-absences corresponds to the number of occurrences in Europe (*N* = 1336), which is the most suitable method for GBMs [43]. Databases with confirmed absence points of *Ae. albopictus* in Europe were not available, only administrative units with absences (e.g., the distribution map of *Ae. albopictus* from the European Centre for Disease Prevention and Control ECDC [38]). However, this information is not sufficient for modelling as the exact locations of absence within these administrative units are missing.

For future projections of climatically suitable areas in the near future (2021–2040) we used data from the Earth system model of the Max Planck Institute for Meteorology (MPI-ESM-lr), downloaded from the website of the Consortium of International Agricultural Research Centers CGIAR program on Climate Change, Agriculture and Food Security (CCAFS) [44]. From the various available emission scenarios based on the Intergovernmental Panel on Climate Change IPCC [45], representative concentration pathway RCP 8.5 was selected as an extreme scenario (radiation force of 8.5 W/m^2^ in 2100 versus 1850) with an expected increase in temperature of about 4.3 °C by the end of the century in comparison to the pre-industrial times [46].

Infections with DENV and CHIKV have been legally notifiable infections in Germany for years, with a general arbovirus notification requirement (including ZIKV) only coming into force in May 2016 [47]. Laboratories have to notify diagnoses of acute infections to local health departments who investigate further information such as travel history. Suitable areas for a likely establishment of *Ae. albopictus* in Germany were combined with the incidence of (potentially viraemic) travel-associated CHIKV and DENV infection cases at the county level over the years 2011–2017 (from the Robert Koch Institute RKI-hosted national-level database on notifiable diseases SURVNET [48]). As a spatial reference for the German counties, data provided by the German Federal Agency for Cartography and Geodesy was used [49]. The data product vg1000-ew also contains the official number of inhabitants per county as of 31 December 2016, which was used to calculate the incidence rate (cases per 100,000 inhabitants). In order to carry out a classification of the hazard potential for virus transmission, first the spatial average of climatic suitability was calculated for each county based on the rasterized output of the GBM. Then, both climatic suitability and incidence rate data were re-scaled to values between 0 and 1, and the two layers were multiplied with each other to gain an estimate of over-all hazard potential per county. Continuous values were divided into three classes using Jenks natural breaks. Seasonal coupling of vector occurrence and viral disease incidences was not considered, since the incidences were summarized for 7 years. All analyses were made using R version 3.4.2 [42].

## 3. Results

### 3.1. Current and Future Climatically Suitable Areas for the Establishment of Aedes albopictus

**Figure 2:**
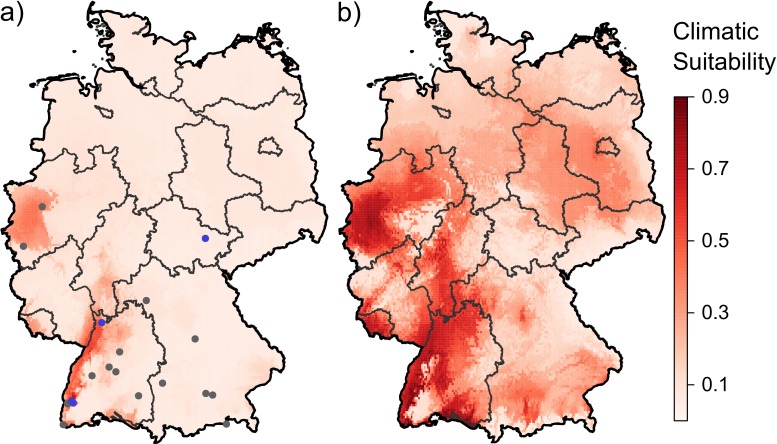
(**a**) Relative climatic suitability for the establishment of *Aedes albopictus* in Germany. Circles: high-precision occurrence records derived from the literature for both single introduction events (grey) and established (overwintering) populations (blue); only the latter were used for modelling. (**b**) Projected future suitable climates for the establishment of *Aedes albopictus* in Germany (near future 2021–2040, climate model mpi-esm-lr, climate scenario RCP 8.5). (**a**,**b**) global climatic dataset worldclim with spatial resolution of 2.5 arcmin (≈5 km). Lines delineate level-2 administrational units (federal states) of Germany.

The federal states of Baden-Württemberg, the Saarland, Rhineland-Palatinate, Hesse and North Rhine-Westphalia current show the highest values of climatic suitability for *Ae. albopictus* (Figure 2a, see Figure S1 for geographical reference). Thus far, established populations (long-term presence, locally reproducing, overwintering) have been found in Baden-Württemberg (Heidelberg, Freiburg) and Thuringia (Jena). The two locations where single specimens of the mosquito have been found in North Rhine-Westphalia lie in an area that is classified as climatically suitable by the model, suggesting that surveillance activities should be intensified in order to avoid unnoticed establishment of populations. The same is currently not true for the locations of introduced specimens in Bavaria and eastern Baden-Württemberg, but this may change sooner rather than later. In the near future (2021–2040), the area climatically suitable for the mosquitoes strongly extends into Germany and reaches high values of suitability all over the western and southern parts of the country, excluding the extreme North-West and low mountain landscapes (Figure 2b). Increasing climatic suitability is found in Lower Saxony, Saxony-Anhalt, Brandenburg, northern regions of Saxony, and the cities of Berlin, and Hamburg, as well as in north-western and southern parts of Bavaria. The altitudinal pattern of Germany is reflected in the temperature and precipitation variables used in our model: *Ae. albopictus* is less likely to establish in higher elevations such as the Black Forest, Swabian Jura hills, the Bavarian forest, Ore Mountains, and the Rothaar Mountains in Germany.

### 3.2. German Counties and Population Showing a Hazard Potential for Autochthonous Transmission of Dengue and Chikungunya Viruses

**Figure 3:**
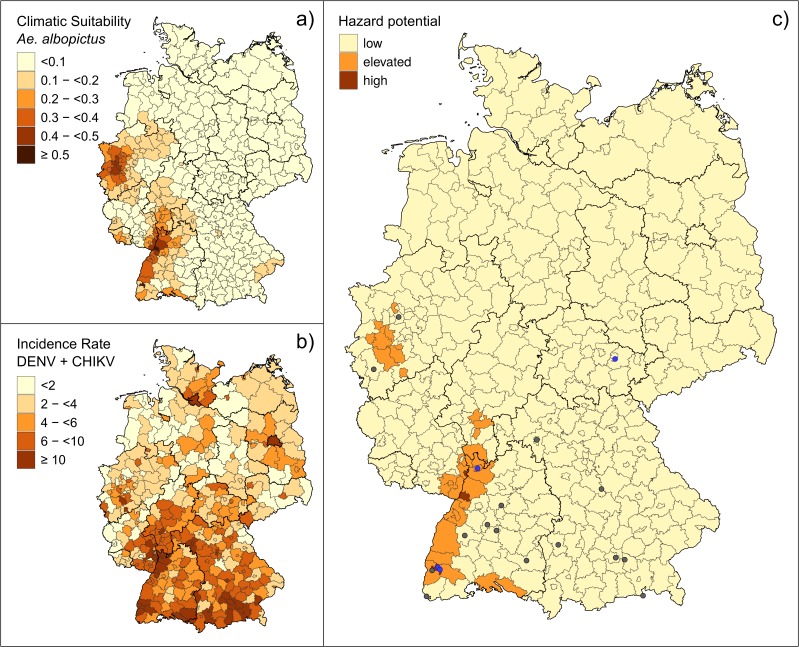
(**a**) Current climatic suitability for the establishment of *Aedes albopictus* in Germany, averaged over the county level in Germany. (**b**) Incidence of (potentially viraemic) travel-associated CHIKV and DENV infection cases at the county level (cases per 100,000 population by county over the years 2011–2017, from the RKI-hosted national-level database on notifiable diseases SURVNET). (**c**) Likelihood of *Aedes albopictus* meeting viraemic returning travellers, potentially leading to transmission, shown as a combination of the modelled climatic suitability for *Aedes albopictus* at the county level, and DENV and CHIKV incidence in returning travellers. Circles: high-precision occurrence records derived from the literature for both single introduction events (grey) and established (overwintering) populations (blue) of *Aedes albopictus*; only the latter were used for modelling. Black and grey lines respectively indicate level-2 and level-3 administrational units (federal states and “Kreis”) of Germany. Administrative units provided by the Federal Agency for Cartography and Geodesy in Germany BKG: GeoBasis-DE / BKG 2013.

When the climatic suitability is averaged on county level, Baden-Württemberg, Hesse and North Rhine-Westphalia current show the highest number of counties and cities climatically suitable for *Ae. albopictus* (Figure 3a).

Autochthonous mosquito-borne DENV and CHIKV cases have not been identified in Germany so far. Travel-associated CHIKV and DENV infections in Germany are most frequently diagnosed in large cities such as Berlin (number of cases 2011-2017: 511), Munich (405), Hamburg (234), Cologne (149), and Frankfurt am Main (114) (Figure 3b, Table S1). Counties in the direct neighbourhoods of these metropolitan areas also show an elevated incidence of DENV and CHIKV cases (Rhein-Neckar-Kreis county (65), Munich county (57), Hannover county (55), and Karlsruhe county (47)). Overall, southern states of Germany appear more affected than others, with the highest incidences found in Bavaria and Baden-Württemberg. DENV cases are more frequent than CHIKV cases.

German counties and cities showing a high hazard potential for autochthonous transmission of *Ae. albopictus*-borne arboviruses in case of further establishment of the vector are the cities of Freiburg im Breisgau, Speyer, and Karlsruhe (Figure 3c, Table S1). The species formed at least two separate reproducing populations in Freiburg that were able to overwinter in 2015–2016 [28,29,50].

An elevated hazard potential of transmission becomes apparent mainly along the Rhine river valley and adjacent portions of its tributaries in Baden-Württemberg, southern parts of Hesse, and North Rhine-Westphalia as far north as Duisburg. Within this category, the cities Mannheim, Cologne, Heidelberg, Frankfurt am Main, and Ludwigshafen, and the counties Karlsruhe, Emmendingen, Rhein-Pfalz-Kreis, Rhein-Neckar-Kreis, and Germersheim show the highest relative values. While most of the counties, cities and districts along the Upper Rhine are potentially covered by mosquito control (Figure S2), no comparable permanent infrastructure exists in the densely populated North Rhine-Westphalia.

Applying the previously described categorization, currently about 0.5 million people are living in Germany in areas that have a high hazard potential for an autochthonous transmission of DENV or CHIKV during the active season of the vector mosquito. In addition, 8.8 million people live in regions showing an elevated hazard potential if vector establishment continuous to progress in climatically suitable areas. Of all these, 1.7 million people live in administrative units that are members of the German Mosquito Control Association.

## 4. Discussion

Mosquito-borne diseases such as DENV, CHIKV and Zika have spread and expanded globally during the last decades. At the same time, an increase of established populations of the competent vector *Ae. albopictus* in Germany creates an emerging health hazard potential for seasonal autochthonous transmission of non-endemic mosquito-borne viral diseases. For the first time, spatially explicit information on DENV and CHIKV incidence found in travellers in Germany is combined with the current climatic suitability for vector establishment. This identification of areas with transmission potential on the county level supports public administrations to effectively plan adequate vector control measures and to intensify surveillance and raise awareness to avoid autochthonous transmission of *Ae. albopictus*-associated arboviruses. Beside the public health hazards, the daytime biter *Ae. albopictus* is also known to be a significant biting nuisance and thus may negatively impact tourism and outdoor activities, leading to economic loss [51].

The limitations of the model approach used here should be taken into account. The strength of a species distribution model depends on the quality and quantity of the occurrence records as well as the environmental data [52]. Aside from further areas rendered suitable by climate change, *Ae. albopictus* is likely not yet occupying all currently suitable areas in Europe. In Southern Europe, *Ae. albopictus* may not yet have been forced to apply its full cold adaptation capacity. In this case, our model would underestimate the potential areas of suitability in Germany. However, we expect only a minor influence on our modelling results due to the large amount of already available data in Europe as well as the good model performance.

To fit our models, environmental variables with a spatial resolution of 2.5 arcmin (≈5 km) are used. This is still quite coarse for an insect species and does not account for suitable microclimatic conditions allowing spatial and temporal windows of opportunity for establishments of *Ae. albopictus*. This was the case in at least one sheltered valley in Thuringia: Jena is one of the most climatically favourable regions in Germany. Reflected solar radiation on the steep slopes and heat storage of the shell limestone are responsible for mild springs, hot summers, long and warm autumns, and mild winters. Due to the warm microclimate, the region near Jena is also called the “Tuscany of the East”. Here, vector monitoring discovered a locally reproducing population of *Ae. albopictus* in 2015 [53], but this area was too small to be recognized as having a suitable climate by the model.

Aside from data-related issues, the choice of model algorithm is a major source of uncertainty in correlative species distribution modelling [54]. To reduce the uncertainty in model projections, we initially used four model algorithms and reduced the set later to the one algorithm that performed best regarding the AUC/TSS and reasonably reflected the observed data.

For the selection of pseudo-absences, the application of different methods influences the modelling results [55]. We used a random pseudo-absence selection with the same number of pseudo-absences as the number of presences [43]. As *Ae. albopictus* continues to spread in Europe and niche shifts are likely [56], we intended to avoid the exclusion of areas likely to be suitable by defining minimum or maximum distances around occurrences for the pseudo-absence selection.

Besides the vector´s climatic suitability, temperature also directly impacts the transmission process of viruses, as the extrinsic incubation period (defined as the period between infection of the insect vector and its ability to transmit the virus to other susceptible hosts) depends on the ambient temperature (see e.g., [57]). Accounting for the extrinsic incubation period can add a temporal aspect in the description of vector-borne disease risk especially in temperate regions.

The numbers of diagnosed and notified cases of DENV and CHIKV infection underestimate the true number of imported (symptomatic and asymptomatic) infections, mostly because not every infected traveller will make use of medical counselling. There still may be residually better access to diagnostics in urban as compared to rural areas (e.g., by easy access to centres for tropical medicine) resulting in an underestimation of hazard in rural areas. At the same time, the notified case numbers overestimate the number of viraemic returnees because a proportion of patients notified as cases is already non-viraemic upon return to Germany. Nevertheless, as an indicator for relative and geographic frequency of import of travel-associated infections, notified infections represent the best available data source. The resulting categories consider the distribution of the available data on travel-associated infections as well as the range of modelled suitability values and observed incidences and thus can serve as an indicator for the spatial patterns of hazard potential.

Long-term mosquito control can reduce the number of invasive mosquito populations and/or mosquito abundance which in turn will lower a potential disease transmission risk. However, currently barriers exist for implementation and expansion of mosquito control and surveillance programs. Unclear responsibilities among the various authorities involved (such as environmental authority, public health authority, and local authorities), and a lack of standardized procedures for monitoring and intervention plans as well as different interpretations of the existing legal basis hamper rapid and successful implementation.

Most counties in Germany are not experienced in getting to terms with establishing container-breeding vector mosquitoes. Until recently, large-scale vector control measures conducted by the German Mosquito Control Association have focused primarily on *Aedes vexans* and similar species that lay their eggs in the moist soils of the floodplains and riparian forests around the Rhine river. After flood events, large areas are treated with *Bacillus thuringiensis israelensis* (BTI) on foot and from helicopters. While this method has proven to be effective against those species, *Ae. albopictus* poses a different challenge. As it prefers small water bodies (such as rain barrels or flower vases) as breeding sites, surveillance and monitoring efforts need to be directed towards different kinds of habitats. Similarly, control of *Ae. albopictus* requires a much more targeted approach. When the first mass development of *Ae. albopictus* in Germany occurred in an allotment garden in Freiburg, control measures included the removal of breeding sites and deployment of BTI tablets. For the future, release of sterilized or *Wolbachia*-infected males has been considered as an additional measure [27].

Vector abundance data is not sufficiently available yet but will improve future model approaches. Assuming at least similar numbers of infected travellers returning to Germany over the upcoming two decades and taking into account the increasing climatic suitability for vector establishment especially in western and southern parts of the country, a further increase in the size of the population at risk can be expected.

## 5. Conclusions

Despite its limitations, the model is an important step forward, because for the first time spatially explicit information about travel-related arbovirus infections in Germany is combined with data on the vector’s climatic suitability. Overall, a more targeted and thus cost-efficient implementation of adequate vector control measures, health surveillance, and awareness raising are supported by the detailed maps provided here. At a national scale, besides Baden-Württemberg, Hesse, and Rhineland-Palatinate, the federal state of North Rhine-Westphalia appears to require the most urgent attention, as several hazard factors come together there. Future approaches should also include additional vector species such as *Aedes japonicus* and the diseases they transmit. The establishment of vectors and introduction of infectious diseases not known yet in Germany is a very dynamic process which requires permanent adaptation and improvement of projections based on new data on vector control, vector occurrence, vector ecology, and arbovirus incidence in returnees.

## Figures and Tables

**Table 1:** European cases of *Aedes albopictus*-associated virus transmission. CHIKV: chikungunya virus; DENV: dengue virus.

Virus	Year	Region	Number of Cases	Reference
CHIKV	2007	Ravenna region, Italy	ca. 200	[12]
CHIKV	2010	Var, France	2	[13]
CHIKV	2014	Montpellier, France	14	[14]
CHIKV	2017	Var, France	9	[15]
CHIKV	2017	Rome and Anzio, Italy	ca. 400	[16]
DENV	2010	Nice, France	2	[17]
DENV	2010	Croatia	1	[18]
DENV	2013	Bouches-du-Rhône, France	1	[19]
DENV	2015	Nîmes, France	7	[20]

## Supplementary Materials

The following are available online at http://www.mdpi.com/1660-4601/15/6/1270/s1, Table S1: Current climatic suitability for the establishment of *Aedes albopictus* in Germany, incidence of travel-associated CHIKV and DENV infections (cases per 100,000 population over the years 2011–2017), and hazard potential classes at the county level, Figure S1: Map of Germany with geographical units (federal states, counties, cities and mountain ranges) mentioned in the main text, Figure S2: Overview about the current situation regarding *Aedes albopictus* in Germany.
