# Supplementary material for: Areas with High Hazard Potential for Autochthonous Transmission of Aedes albopictus-Associated Arboviruses in Germany
Source: Int J Environ Res Public Health. 2018 Jun 15;15(6):1270. doi: 10.3390/ijerph15061270 (PMC6025521; doi:10.3390/ijerph15061270)
Supplement: Supplementary file 1 [file ijerph-15-01270-s001.zip › Figure_S2.pdf]

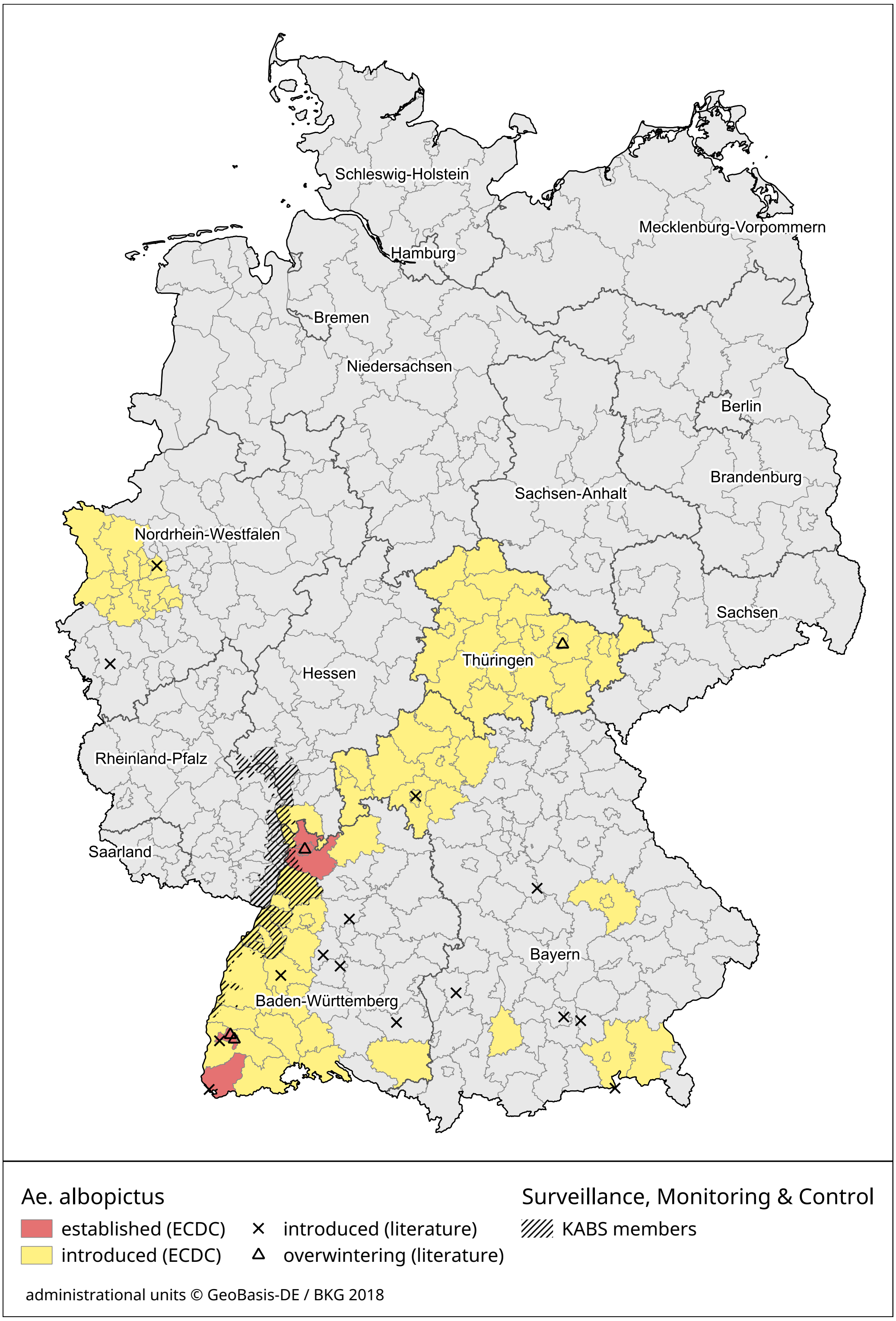

The figure gives an overview about the current situation regarding *Aedes albopictus* in Germany.

The colored areas give the status according to the European Centre for Disease Prevention and Control (ECDC) for German districts ("Landkreis" or "Stadtkreis", equivalent to NUTS level 3 on a European scale) [1]. Precise locations or data sources are not available for these areas.

Crosses and triangles show point locations that are publically available from the scientific literature (2008–2018) [2-5]. Precision varies from precise site descriptions to rough maps.

Hatched areas show municipalities and districts that are members of the German Mosquito Control Association KABS [6]. The Federal States of Baden-Württemberg and Rheinland-Pfalz support KABS as well. Neither the trapping locations of the currently running CuliMo project nor data from the Mueckenatlas citizen science project [7] are publicly available yet.

References

1. European Centre for Disease Prevention and Control. Mosquito maps. Available online: https://ecdc.europa.eu/en/disease-vectors/surveillance-and-disease-data/mosquito-maps (accessed on 2 May 2018).
2. Kraemer, M.U.G.; Sinka, M.E.; Duda, K.A.; Mylne, A.Q.N.; Shearer, F.M.; Barker, C.M.; Moore, C.G.; Carvalho, R.G.; Coelho, G.E.; Van Bortel, W.; et al. The global distribution of the arbovirus vectors *Aedes aegypti* and *Ae. albopictus*. *Elife* **2015,** *4*, e08347, 10.7554/eLife.08347.
3. Pluskota, B.; Jöst, A.; Augsten, X.; Stelzner, L.; Ferstl, I.; Becker, N. Successful overwintering of *Aedes albopictus* in Germany. *Parasitol Res* **2016,** *115* (8), 3245-3247, 10.1007/s00436-016-5078-2.
4. Walther, D.; Scheuch, D.E.; Kampen, H. The invasive Asian tiger mosquito *Aedes albopictus* (Diptera: Culicidae) in Germany: Local reproduction and overwintering. *Acta Trop* **2017,** *166*, 186-192, 10.1016/j.actatropica.2016.11.024.
5. Kampen, H.; Schuhbauer, A.; Walther, D. Emerging mosquito species in Germany-a synopsis after 6 years of mosquito monitoring (2011-2016). *Parasitol Res* **2017,** *116* (12), 3253-3263, 10.1007/s00436-017-5619-3.
6. Die Mitglieder der KABS e.V. Available online: http://www.kabsev.de/1/1_2/1_2_4/index.php (accessed on May 9, 2018).
7. Mückenatlas. Available online: https://www.mueckenatlas.de/ (accessed on May 9, 2018).
