# Supplementary material for: Areas with High Hazard Potential for Autochthonous Transmission of Aedes albopictus-Associated Arboviruses in Germany
Source: Int J Environ Res Public Health. 2018 Jun 15;15(6):1270. doi: 10.3390/ijerph15061270 (PMC6025521; doi:10.3390/ijerph15061270)
Supplement: Supplementary file 1 [file Figure_S1.pdf › Figure_S1.pdf]

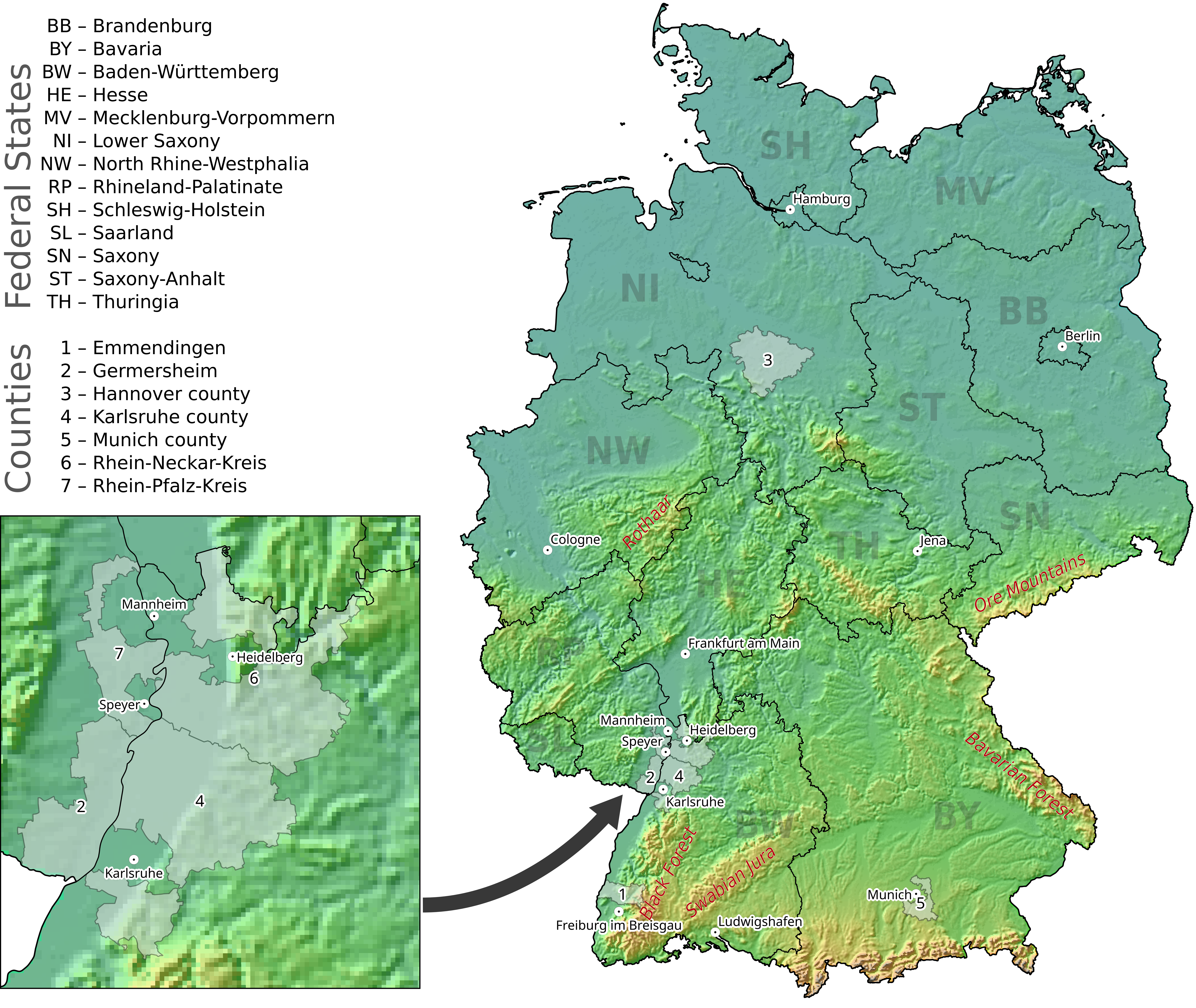

Overview map of Germany including cities, counties and mountain ranges mentioned in the main text, as well as all (non-city) Federal States. Administraional units and digital elevation model © GeoBasis-DE / BKG 2018.
